# Effect of shade and limit feeding in growing beef heifers during periods of heat stress

**DOI:** 10.1093/tas/txae161

**Published:** 2024-11-19

**Authors:** Zachary L DeBord, Zachary M Duncan, Madison G Pflughoeft, Kyler J Suhr, William C Ellis, William R Hollenbeck, Sean P Montgomery, Tyler J Spore, Evan C Titgemeyer, Dale A Blasi, Anthony J Tarpoff

**Affiliations:** Department of Animal Sciences and Industry, Kansas State University, Manhattan, KS 66506, USA; Department of Animal Sciences and Industry, Kansas State University, Manhattan, KS 66506, USA; Department of Animal Sciences and Industry, Kansas State University, Manhattan, KS 66506, USA; Department of Animal Sciences and Industry, Kansas State University, Manhattan, KS 66506, USA; Department of Animal Sciences and Industry, Kansas State University, Manhattan, KS 66506, USA; Department of Animal Sciences and Industry, Kansas State University, Manhattan, KS 66506, USA; Corn Belt Livestock Services, Papillion, NE 68046, USA; Innovative Livestock Services, Inc., Great Bend, KS 67530, USA; Department of Animal Sciences and Industry, Kansas State University, Manhattan, KS 66506, USA; Department of Animal Sciences and Industry, Kansas State University, Manhattan, KS 66506, USA; Department of Animal Sciences and Industry, Kansas State University, Manhattan, KS 66506, USA

**Keywords:** growing cattle, heat stress, limit feeding, shade, water

## Abstract

Experiments were conducted during the summers of 2021 and 2022 to evaluate the effects of feeding strategy and shade on growth performance, animal comfort, water usage, apparent diet digestibility, and ruminal fermentation characteristics of growing heifers during periods of heat stress. In Exp. 1, 852 heifers (initial body weight [BW] = 251 ± 13 kg) were assigned to one of 4 treatments: high-energy diet limit-fed at 2.2% of BW (dry matter [DM] basis; LIM) or high-roughage diet fed for ad libitum intake (ADLIB) with shade (SH) or without shade (NSH). Pen BWs were measured on day 0, weekly from days 14 to 84, day 90, and day 97. Pen weights were used to adjust weekly intakes of LIM. Refusals for ADLIB were targeted at 5% of feed consumed the previous day. Following the 90-d feeding period, a gut-fill equilibration diet was fed to all cattle at 2.5% of BW (DM basis) for 7 d to balance differences in gut-fill between dietary treatments. Dry matter intake was lesser (*P* < 0.01) for LIM compared with ADLIB. Average daily gain (ADG) and gain:feed (G:F) were greater (*P* < 0.01) for LIM compared with ADLIB. In addition, ADG and G:F were greater (*P* < 0.01) for SH compared with NSH. Water usage was less (*P* < 0.01) for SH heifers compared with NSH and was also less (*P* < 0.01) for LIM compared with ADLIB. Mean panting scores were lower (*P* < 0.01) for SH compared with NSH and LIM compared with ADLIB. In Exp. 2, 16 heifers (initial BW = 254 ± 22 kg) were arranged in 4 replicated 4 × 4 Latin squares to evaluate treatments from Exp. 1. Apparent total tract digestibility of DM and organic matter was greater (*P* < 0.01) for LIM compared with ADLIB. Ruminal pH was more acidic (*P* = 0.02) for LIM compared with ADLIB. Shade did not affect (*P* ≥ 0.68) apparent diet digestibility; however, ruminal pH was greater (*P* < 0.01) for SH compared with NSH. In conclusion, LIM improved feed efficiency, reduced mean panting score, and reduced water usage compared with ADLIB. In addition, SH improved growth performance, reduced water usage, and improved animal comfort during periods of heat stress.

## Teaser

Limit feeding a high-energy diet or providing shade to growing heifers during periods of heat stress can improve growth performance and animal comfort while subsequently reducing water usage.

## Introduction

Periods of elevated heat load can negatively affect the productivity and animal welfare of beef cattle (Edwards-Calloway, 2021). In extreme instances, elevated temperatures paired with high relative humidity and solar radiation can result in mortality losses in livestock systems. St. Pierre et al. (2003) quantified the economic losses to the beef industry that resulted from heat stress to be approximately $369 million/yr. These losses included poor reproductive performance, reduced weight gains, and beef cattle mortality. As abnormal heat-stress events continue to occur, mitigation strategies may need to be implemented to improve livestock efficiency and animal comfort. Previous research has demonstrated that providing shade to beef cattle during periods of heat stress can improve growth performance, animal comfort, and reduce water consumption (Morrison, 1983; Beede and Collier, 1986; Edwards-Calloway et al., 2021).

Another potential strategy to mitigate heat stress is to alter the composition of the diet. Newly received cattle are traditionally fed a high-roughage diet for ad libitum intake during the growing period. Previous research demonstrated that limiting feeding a high-energy diet during the growing period can improve feed efficiency without detrimental effects on growth performance or health (Spore et al., 2018, 2019; Scilacci et al., 2024). Reducing dry matter (DM) intake may modulate heat production, which could potentially improve animal comfort and performance (Mader and Davis, 2004). To our knowledge, no studies have evaluated limit-fed high-energy diets for growing cattle alongside shade. The objectives of this study were to evaluate the use of shade, feeding strategy, and their interaction on performance, animal comfort, water usage, apparent diet digestibility, and ruminal fermentation characteristics during periods of heat stress.

## Materials and Methods

Two experiments were conducted concurrently between May and September of 2021 and 2022 at the Kansas State University Beef Stocker Unit. All procedures involving the use of animals were approved by Kansas State University Institutional Animal Care and Use Committee.

### Experiment 1: Receiving Trial

Over a 2-yr period, 852 predominantly black-hided crossbred heifers (initial body weight [BW] = 251 ± 13 kg) were purchased from Kansas, Iowa, and Missouri and transported to the Kansas State University Beef Stocker Unit. In year 1, 413 heifers (246 ± 15 kg) were received on May 19, May 26, and June 19, 2021. In year 2, 439 crossbred heifers (246 ± 15 kg) were received on May 24, May 25, and June 4, 2022. A randomized block design with a 2 × 2 factorial arrangement was used to determine the influence of feeding strategy (limit-fed vs. ad libitum) and shade (shade vs. no shade) on growth performance and water usage. This created 4 treatments that included: limit-fed with shade, limit-fed with no shade, ad libitum with shade, and ad libitum with no shade. Upon arrival, heifers were blocked by truckload (5/yr), stratified by individual arrival weight within the block, and assigned to pens containing 9 to 12 heifers. Each block contained 8 pens. Within the block, pens were assigned to 1 of 4 treatments for a total of 40 pens and 10 replications per treatment per year for a total of 80 pens and 20 replications per treatment.

Experimental diets are presented in Table 1. Dietary treatments included a high-energy diet formulated to provide 1.32 Mcal of net energy for gain (NEg)/kg DM limit-fed at 2.2% of BW once daily (LIM) and a high-roughage diet formulated to provide 0.99 Mcal NEg/kg of DM fed for ad libitum intake (ADLIB). Animals were fed once daily beginning at 0700 hours using a Roto-Mix feed wagon (Model 414-14B, Dodge City, KS). Bunks were observed prior to feeding to estimate ad libitum intake. Refusals for ad libitum diets were targeted at 5% of DM consumed the previous day. Using a pen scale (Rice Lake Weighing Systems, Rice Lake, WI), pen BW was measured on day 0, weekly from days 14 to 84, day 90, and day 97. Pen weights were used to adjust feed delivery for LIM diets and calculate animal performance. At the completion of the 90-d feeding period, a gut-fill equilibration diet (Table 1) was fed for 7 d to balance for differences in gastrointestinal tract fill between LIM and ADLIB heifers. The gut-fill equilibration diet was offered at 2.5% of BW once daily (DM basis) and formulated to provide 1.15 Mcal NEg/kg DM.

**Table 1.** Composition of experimental diets

	Diet^1^		
	ADLIB	LIM	GUTFILL
Ingredient, % DM			
Dry-rolled corn	8.6	38.8	23.8
Supplement^2^	6.4	8.2	6.9
Wet corn gluten feed^3^	40.0	40.0	40.7
Alfalfa hay	22.5	6.5	14.2
Warm-season grass hay	22.5	6.5	14.4
*Experiment 1*			
Nutrient composition, % DM			
Dry matter, % as is	77.1	76.0	76.4
Organic matter	92.4	94.1	93.5
Crude protein	16.0	15.2	15.3
Neutral detergent fiber	41.0	26.2	33.7
Acid detergent fiber	20.7	9.7	15.1
Calcium	1.0	0.9	0.8
Phosphorus	0.6	0.6	0.6
*Experiment 2*			
Nutrient composition, % DM			
Dry matter, % as is	77.9	77.0	—
Organic matter	92.6	94.2	—
Crude protein	16.3	15.4	—
Neutral detergent fiber	40.7	26.1	—
Acid detergent fiber	20.6	9.7	—
Calcium	1.0	0.8	—
Phosphorus	0.6	0.6	—

^1^ADLIB: Formulated to provide 0.99 Mcal of NEg/kg of DM fed for ad libitum intake; LIM: 1.32 Mcal of NEg/kg of DM fed at 2.2% of BW (DM basis); GUTFILL: gut-fill equilibration diet fed a 2.5% of BW (DM basis) from days 90 to 97.

^2^Supplement pellet formulated to contain (DM basis) 8.4% Ca, 5% NaCl, and 360 mg/kg monensin. Supplement ingredients: 72.15% wheat middlings, 22.0% calcium carbonate, 5% NaCl, 0.35% soybean oil, 0.18% Rumensin 90 (Elanco, Greedfield, IN), 0.11% zinc sulfate, 0.08% manganese (Mn) sulfate (32% Mn), 0.06% vitamin E premix (500,000 IU/kg), 0.05% copper sulfate, 0.01% selenium premix (0.99% Se), 0.007% ethylenediamine dihydriodide (EDDI) premix (11.4% EDDI), and 0.004% vitamin A (650,000 IU/g).

^3^Sweet Bran (Cargill Corn Milling, Blair, NE).

Prior to the experiment, 2 shade structures (12.19 m × 12.19 m) per block of 8 pens were randomly assigned to cover 2 pens per structure; for the 2 pens under a common shade structure, 1 pen was fed each of the dietary treatments. Therefore, 4 pens per block were shaded (SH), and the remaining 4 were nonshaded (NSH). Shade structures were 4.3 m tall, blocked 70% of sunlight, and provided 7.2 ± 0.6 m^2^ of shade per animal (Strobel Manufacturing Inc., Clarks, NE). Pens were soil-surfaced and of equal size (9.1 × 15.2 m) with bunks of 9.1 m in length attached to a 3.6-m concrete apron.

On arrival (day −1), cattle were individually weighed, assigned a visual identification ear tag and an electronic identification ear tag, and provided 2.27 kg of warm-season grass hay per animal (DM basis) with ad libitum access to water. On day 0, heifers were administered a 7-way clostridial vaccine (Vision 7 with SPUR, Merck Animal Health, Madison, NJ), a modified-live vaccine to protect against infectious bovine rhinotracheitis, parainfluenza, and bovine viral diarrhea types 1 and 2 (Vista Once SQ, Merck Animal Health), and an anti-parasitic drench (Valbazen, Zoetis Animal Health, Parsippany-Troy Hills, NJ).

All heifers were observed twice daily for signs of depression, lameness, nasal or ocular discharge, and morbidity. Heifers exhibiting a Clinical Illness Score (CIS) greater than 1 (1 = normal and healthy, 2 = slightly ill with mild depression and gauntness, 3 = moderately ill, with severe depression, labored breathing, and ocular or nasal discharge, 4 = severely ill, near death, or little response to human approach) were removed from the pen for further evaluation.

Heifers exhibiting a rectal temperature of ≥40 °C and CIS ≤ 2 or with a rectal temperature ≤40 °C and CIS ≥ 3 were treated according to the study health protocol. Heifers treated for the first time received florfenicol (0.13 mL/kg BW; Norfenicol, Norbrook Labs, Lenexa, KS), second treatments were enrofloxacin (0.12 mL/kg BW; Enroflox 100, Norbrook Labs), and third treatments consisted of oxytetracycline (0.10 mL/kg BW; Pro LA 300, Norbrook Labs). Treatments were administered subcutaneously. Heifers treated 3 times were designated as chronically ill and removed from the study.

Individual feed ingredient samples were collected weekly. A portion of each ingredient sample was dried in a forced-air oven at 105 °C for 48 h and was used to adjust diet DM. The remaining feed ingredient sample was immediately frozen at −20 °C. At the completion of the feeding period, feed ingredient samples were thawed and composited.

### Rumination and Activity

In year 1, all animals received a 3-axial accelerometer on day 0 (Allflex Livestock Intelligence, Madison, WI). Tags continuously recorded activity and rumination, which were summarized in 2-h increments and compiled for daily activity and rumination measurements (min/d). Rumination measurements quantify rumination and mastication. Activity measurements quantify all animal activity, not including rumination. Data from the first 10 d were removed to allow accelerometer readings to stabilize. Due to availability, accelerometers were not applied in year 2.

### Weather Measurements

Weather conditions were continuously measured using a Davis weather station (Vantage Pro 2, Davis Instruments Corporation, Hayward, CA) located in the center of the feedlot. Weather conditions measured included: ambient temperature (°C), relative humidity (%), wind speed (km/h), and solar radiation (W/m^2^). Temperature humidity index (THI) was calculated using the equation adapted from Thom (1959) where THI = 0.8 × ambient temperature + ((relative humidity/100) × (ambient temperature − 14.3)) + 46.4.

### Animal Comfort Index

Criteria used for panting score evaluation are presented in Table 2. To determine the effects of shade on animal comfort, heifers were evaluated at 0930, 1330, and 1730 hours on days when the temperature humidity index was estimated to be greater than 74 using the United States Meat Animal Research Center Animal Comfort Index. Using a method adapted from Guaghan et al. (2008; Table 2) individual panting score was determined using respiration rate and breathing conditions. Three heifers per pen were selected randomly at each time point within each pen. The 3 values were averaged for each time point and each day to obtain a mean panting score for each pen on an hourly and daily basis.

**Table 2.** Panting score evaluation system^1^

Score	Breathing conditions
0	No panting. Respiration < 60 breaths/min
1.0	Slight panting, mouth closed, no drool, easy to see chest movement. Respiration: approximately 60 to 90 breaths/min.
1.5	Moderate panting, no drool present, easy to see chest movement, mouth closed. Respiration: approximately 60 to 90 breaths/min.
2.0	Fast Panting, drool present, mouth closed. Respiration: approximately 90 to 120 breaths/min.
2.5	Fast Panting, drool present, occasional mouth panting. Respiration: approximately 90 to 120 breaths/min.
3.0	Occasional open mouth panting, excessive drooling, neck extended, head held up. Respiration: approximately 120 to 150 breaths/min.
3.5	Open mouth panting, excessive drooling, tongue slightly extended or occasionally extended for short periods. Respiration: approximately 120 to 150 breaths/min.
4.0	Open mouth with extended tongue for a prolonged period, excessive drooling, neck extended, and head up. Respiration may decrease. Respiration: approximately 120 to 150 breaths/min.
4.5	Open mouth with extended tongue for a prolonged period, excessive drooling, neck extended, head up, visible breaths from the flank, drooling may be ceased. Respiration may decrease. Respiration: approximately 120 to 150 breaths/min.

^1^Adapted from Gaughan et al. (2008).

### Water Usage

Water usage was measured using iPERL water meters (SENSUS, Morrisville, NC) connected to individual automatic waterers (Lil’ Spring 3000; Miraco Livestock Water Systems, Grinnell, IA) for each pen. The total water usage for each pen was divided by the number of heifers per pen to determine the daily water usage per heifer.

### Net Energy Calculations

Performance data were used to calculate dietary net energy for maintenance (NEm) and NEg concentrations as described by Galyean (2019) using NRC (1996) nutrient requirement equations. Prior to calculating dietary net energy concentrations, a 4% shrink was applied to initial (day 0) and final (day 97) BWs.

### Experiment 2—Feed Intake, Apparent Diet Digestibility, and Ruminal Fermentation Characteristics

Over a 2-yr period, 16 ruminally cannulated black-hided crossbred heifers (initial BW = 254 ± 22 kg) were used in 4 replicated 4 × 4 Latin squares. Eight heifers (initial BW = 254 ± 29 kg) were used in year 1, and 8 heifers (255 ± 17 kg) were used in year 2. Experimental unit was animal within the period, and treatments were identical to Exp. 1. Ruminal pH data from period 1 of year 1 were removed due to a pH meter failure. Ruminal pH, ruminal volatile fatty acid (VFA), and ruminal ammonia data from 1 LIM NSH heifer and 1 ADLIB NSH heifer in period 3 of year 1 were removed because ruminal contents were lost prior to collection. In addition, 4 d of diet adaptation was added to period 4 of year 2 because of scheduling. All other procedures for Exp. 2 were identical for both years.

Prior to the start of the experiment, 8 pens equal in size were constructed. One group of 4 pens shared 2 shade structures, each 3.7 m × 4.9 m (JobSmart, Tractor Supply, Brentwood, TN) to provide 8.9 m^2^ of shade per animal. Each structure was 3.05 m tall and blocked 70% of sunlight. The second group of 4 pens did not contain shade. Cattle were fed diets from Exp. 1 (Table 1) once daily at 1000 hours. Each morning, feed for both experiments was mixed using a Roto-Mix feed wagon, and the beginning of each load was removed and fed to heifers in Exp. 2.

The experiment consisted of 4 consecutive 15-d periods. Each period included 10 d of diet adaptation, 4 d of fecal collection, and 1 d of ruminal fluid collection. To estimate fecal output and determine apparent digestibility, 10 g of chromium oxide (Cr_2_O_3_) was placed through the rumen cannula below the hay mat on days 4 to 14 using a 24-mL gelatin capsule (Torpac, Fairfield, NJ). On days 11 to 14, fecal samples were collected from each animal at 8-h intervals postfeeding. Fecal sampling times advanced 2 h each day so that each 2-h interval in a 24-h period was represented. Immediately following collection, 100 g of each fecal sample was composited within animal within each period. Individual feed ingredient samples were collected on days 10 to 14 and immediately frozen at −20 °C. At the conclusion of each period, individual feed ingredient samples were composited.

On day 15, 0-h ruminal digesta samples (approximately 1,500 mL) were collected prior to feeding. To achieve a representative sample, digesta was collected from the dorsal, ventral, cranial, and caudal sacs of the rumen. Following 0-h sampling, 3 g of cobalt-EDTA (containing 0.4 g cobalt) dissolved in 200 mL of deionized water was dosed via rumen cannula to estimate liquid passage rate. Heifers were fed, and ruminal digesta samples were collected at 2, 4, 6, 8, 12, 18, and 24 h after feeding. Following collection, digesta samples were mixed thoroughly and then strained through 8 layers of cheesecloth. The pH or the strained ruminal fluid was measured using a portable pH meter (PH8500 pH/mV Meter, Apera, Columbus, OH). Subsequently, strained ruminal fluid (1 mL) was pipetted into four 2-mL microcentrifuge tubes containing 250 µL of 25% (wt/vol) *m*-phosphoric acid. To determine ruminal cobalt concentrations, 15 mL of strained ruminal fluid was pipetted into 20-mL scintillation vials 2, 4, 6, 8, 12, 18, and 24 h post-feeding. Following collection, all samples were immediately frozen at −20 °C.

### Laboratory Analysis

At the completion of the experiment, composite feed ingredient samples for Exp. 1 and 2 and composite fecal samples were sent to a commercial laboratory (SDK Laboratories, Hutchinson, KS) for analysis. Samples were analyzed for DM, organic matter (OM; 100—ash; AOAC International, 1999), crude protein (N × 6.25; Van Soest, 1991), neutral detergent fiber (NDF; Van Soest, 1991), acid detergent fiber (ADF; Van Soest et al., 1991), calcium (Bowers and Rains, 1988), and phosphorus (AOAC International, 1999).

Ruminal fluid samples collected for VFA and ammonia analyses were centrifuged at 17,000 × *g* for 30 min at 4 °C. Volatile fatty acid concentrations of the supernatant were analyzed using gas-liquid chromatography as described by Vanzant and Cochran (1994). With this analysis, isovalerate included 2-methylbutyrate and 3-methylbutyrate. Ruminal ammonia concentrations of the supernatant were determined according to the procedures described by Broderick and Kang (1980).

Samples retained for ruminal cobalt analysis were centrifuged at 25,000 × *g* for 25 min at 4 °C. Cobalt concentrations of the supernatant and the initial cobalt-EDTA dose were measured using atomic absorption spectrophotometry. The nonlinear procedure of SAS (SAS 9.4, SAS Inst. Inc, Cary, NC) was used to regress the natural logarithm of ruminal cobalt concentrations against time using samples collected from 2, 4, 6, 8, 12, and 18 h post-feeding. Liquid passage rate was determined as the additive inverse of the slope of the regression. Ruminal liquid volume was calculated by dividing the amount of cobalt dosed by the 0-h intercept of the regression after back-converting the intercept to concentration.

To determine apparent diet digestibility, approximately 0.5 g of dried fecal material that had been ground using a 1-mm screen was placed in a muffle oven at 600 °C for 2 h. Chromium concentrations of each sample were determined using atomic absorption spectrophotometry following solubilization using procedures described by Williams et al. (1962). Fecal chromium concentrations were used to determine total fecal output and apparent diet digestibility as described by Cochran and Galyean (1994).

### Statistical Analyses

In Exp. 1, all data were analyzed using the MIXED procedure of SAS with a pen as the experimental unit. For growth performance, feed intake, dietary net energy concentrations, and water usage, the model included fixed effects of diet, shade, diet × shade, year, diet × year, shade × year, diet × shade × year, and a random effect of block within year. For panting score data, the model included fixed effects of diet, shade, diet × shade, time, diet × time, shade × time, and diet × shade × time. Random effect was block within a year. Panting score data were also averaged over the duration of the study by hour and analyzed with the models described for performance.

Rumination and activity data were analyzed as repeated measures. The model included fixed effects of diet, shade, diet × shade, hour, diet × hour, shade × hour, diet × shade × hour with hour as the repeated measure using pen as the subject; block was included as a random effect. The covariance structure for rumination and activity was spatial power as determined by better-fit characteristics of Akaike information criterion (AIC).

In Exp. 2, all data were analyzed using the MIXED procedure of SAS with animal within the period as the experimental unit. Feed intake, apparent diet digestibility, liquid passage rate, and ruminal liquid volume were analyzed with diet, shade, diet × shade, year, diet × year, shade × year, diet × shade × year, and period within a year as fixed effects and animal within year as a random effect. Ruminal ammonia and VFA concentrations were analyzed using repeated measures. The model included fixed effects of diet, shade, diet × shade, hour, diet × hour, shade × hour, diet × shade × hour, diet × year, shade × year, shade × diet × year, and period within a year. Animal within year served as a random effect. Covariance structure was spatial power according to AIC statistics. For all analyses, significance was declared at *P* ≤ 0.05 and tendencies at *P *≤ 0.10.

## Results and Discussion

### Weather Conditions

Weather conditions recorded during the summer of 2021 and 2022 are presented in Table 3 and 4, respectively. Mader et al. (2006) reported adjustments for the livestock weather safety index which classifies the risk of heat stress into 4 categories based on THI. A THI less than 74 is considered normal, 74 to 79 an alert, 79 to 84 a danger, and greater than 84 is an emergency heat-stress condition. Weather conditions during the summers of 2021 and 2022 were similar; average THI was 76 ± 7.9 and 76 ± 6.7 for 2021 and 2022, respectively. In year 1, THI was 74 or greater for 90 d and reached a maximum of 96.6. In year 2, THI was 74 or greater for 78 d and reached a maximum of 87.9. These data demonstrate that heat-stress conditions were present and were likely sufficient to evaluate heat-stress mitigation strategies.

**Table 3.** Weather conductions collected at the Kansas State Beef Stocker unit during summer 2021

Item^1^	May	June	July	August	September	Overall
Temperature, °C						
Mean	19.9 ± 4.2	28.2 ± 4.5	28.3 ± 4.0	28.9 ± 4.2	25.8 ± 4.4	26.6 ± 5.2
Maximum	30.6	38.3	37.2	36.7	35.0	38.3
Minimum	8.9	15.0	18.3	17.2	10.0	8.9
Relative humidity, %						
Mean	76.7 ± 13.9	66.4 ± 7.0	68.4 ± 6.9	69.8 ± 5.6	61.4 ± 8.9	68.0 ± 9.7
Maximum	96.0	88.0	79.0	81.0	74.0	96.0
Minimum	33.0	32.0	25.0	26.0	26.0	25.0
Wind speed, km/h						
Mean	12.4 ± 6.3	9.4 ± 6.2	9.2 ± 4.8	12.1 ± 6.0	12.2 ± 6.2	10.9 ± 6.1
Maximum	30.6	27.4	29.0	35.4	37.0	37.0
Minimum	0	0	0	0	0	0
Solar radiation, W/m^2^						
Mean	310 ± 236	510 ± 265	498 ± 254	456 ± 258	382 ± 242	445 ± 262
Maximum	1,186	1,104	1,201	1,060	902	1,201
Minimum	0	15	1	0	0	0
THI^2^						
Mean^3^	66.5 ± 6.3 (1)	78.2 ± 6.8 (24)	78.7 ± 6.3 (27)	79.8 ± 6.7 (26)	74.3 ± 6.5 (12)	76.1 ± 7.9 (90)
Maximum	77.7	93.4	93.1	92.1	96.6	96.6
Minimum	49.2	58.7	63.4	61.8	52.6	49.2

^1^Weather data collected by Davis weather station (Vantage Pro 2, Davis Instruments Corporation, Hayward, CA).

^2^THI equation adapted from Thom (1959). THI = 0.8 × ambient temperature + ((relative humidity/100) × (ambient temperature − 14.3)) + 46.4.

^3^Numbers within parentheses denote days during the trial when THI reached above 74.

**Table 4.** Weather conductions collected at the Kansas State Beef Stocker unit during summer 2022

Item^1^	May	June	July	August	September	Overall
Temperature, °C						
Mean	21.1 ± 7.1	27.0 ± 4.9	28.4 ± 4.4	28.7 ± 4.5	27.3 ± 4.1	27.5 ± 5.2
Maximum	31.7	36.7	38.3	37.8	33.3	38.3
Minimum	11.1	13.9	17.8	18.9	16.1	11.1
Relative humidity, %						
Mean	67.9 ± 14.3	64.2 ± 13.4	62.7 ± 14.6	59.2 ± 13.6	55.2 ± 15.2	62.1 ± 14.3
Maximum	93.0	90.0	87.0	86.0	87.0	93.0
Minimum	33.0	32.0	25.0	23.0	28.0	23.0
Wind speed, km/h						
Mean	19.2 ± 11.5	11.2 ± 7.1	9.8 ± 6.0	9.2 ± 5.2	9.1 ± 3.6	10.7 ± 7.1
Maximum	49.9	46.7	32.2	27.4	17.7	49.9
Minimum	0.0	0.0	0.0	0.0	0.0	0.0
Solar radiation, W/m^2^						
Mean	377 ± 291	506 ± 275	456 ± 273	467 ± 267	440 ± 245	467 ± 274
Maximum	1,015	1,112	1,174	955	926	1,174
Minimum	0	0	4	0	2	0
THI^2^						
Mean^3^	67.0 ± 9.92 (3)	75.9 ± 6.5 (18)	77.7 ± 5.3 (26)	77.6 ± 5.4 (28)	75.0 ± 4.7 (3)	76.2 ± 6.7 (78)
Maximum	80.3	87.0	87.9	87.4	81.5	87.9
Minimum	52.5	57.2	62.4	63.0	60.8	52.5

^1^Weather data collected by Davis weather station (Vantage Pro 2, Davis Instruments Corporation, Hayward, CA).

^2^THI equation adapted from Thom (1959). THI = 0.8 × ambient temperature + ((relative humidity/100) × (ambient temperature − 14.3)) + 46.4.

^3^Numbers within parentheses denote days during the trial when THI reached above 74.

## Experiment 1: Receiving Trial

### Health

No diet × shade interactions (*P* = 0.82) or main effects of diet (*P* = 0.13) or shade (*P* = 0.25; data not shown) were observed for the proportion of heifers treated once for respiratory illness. Overall, 2 ADLIB NSH, 5 LIM NSH, 4 ADLIB SH, and 8 LIM SH heifers were treated once for respiratory illness. In addition, 2 ADLIB NSH, 3 LIM NSH, 1 ADLIB SH, and 5 LIM SH heifers were treated twice and 3 ADLIB NSH, 2 LIM NSH, and 1 LIM SH heifers were treated thrice for respiratory illness. No mortality was observed.

### Growth Performance

Performance data are presented in Table 5. Only a few significant interactions between treatments and year were observed (Supplementary Table 1), and none were considered to materially alter the interpretation of the data; thus, they are not discussed. A diet × shade interaction (*P* < 0.01; Table 5) was observed for dry matter intake (DMI) from days 0 to 90 and days 0 to 97 where DMI was greatest for SH ADLIB heifers, intermediate for NSH ADLIB heifers, and least for SH LIM and NSH LIM heifers. Greater DMI (*P* < 0.01) for SH ADLIB compared with NSH ADLIB is in agreement with previous research that also demonstrated improvements in DMI when shade was provided (Mitlöhner et al., 2001, 2002; Gaughan et al., 2010). By design, DMI was greater (*P* < 0.01) for ADLIB compared with LIM; however, because limit-fed heifers were fed at 2.2% of BW daily (DM basis), DMI was similar (*P* = 0.11) between SH LIM and NSH LIM. Gain-to-feed (G:F) from days 0 to 90 tended (diet × shade: *P* = 0.07) to be greatest for SH LIM, intermediate for NSH LIM, and least for SH ADLIB and NSH ADLIB. The trend for greater G:F for SH LIM compared with NSH LIM was associated with numerically greater ADG for SH LIM compared with NSH LIM. No other diet × shade interactions (*P* ≥ 0.28) were observed for BW, average daily gain (ADG), G:F, water usage, or panting scores; therefore, the main effects of diet and shade will be discussed.

**Table 5.** Effect of feeding strategy and shade on heifer performance and water usage during periods of heat stress

	Treatment^1^							
	No Shade		Shade			*P*-value		
Item,	ADLIB	LIM	ADLIB	LIM	SEM^2^	Diet	Shade	D × S
Number of pens	20	20	20	20				
Number of animals	214	213	215	210				
Body weight, kg								
Day 0	252	251	250	251	4.2	0.86	0.05	0.66
Day 14	277	272	278	272	4.5	<0.01	0.57	0.57
Day 90	357	351	364	358	5.8	<0.01	<0.01	0.71
Day 97	357	364	365	369	5.6	<0.01	<0.01	0.73
Average daily gain, kg/d								
Days 0 to 14	1.82	1.46	2.00	1.52	0.143	<0.01	0.05	0.28
Days 0 to 90	1.17	1.11	1.27	1.20	0.038	<0.01	<0.01	0.37
Days 0 to 97	1.09	1.16	1.18	1.22	0.035	<0.01	<0.01	0.35
Dry matter intake, kg/d								
Days 0 to 14	6.12	5.37	6.15	5.35	0.109	<0.01	0.83	0.59
Days 0 to 90	9.18^b^	6.78^c^	9.78^a^	6.82^c^	0.174	<0.01	<0.01	<0.01
Days 0 to 97	9.17^b^	6.93^c^	9.74^a^	6.98^c^	0.170	<0.01	<0.01	<0.01
Days 90 to 97	9.43	9.42	9.54	9.51	0.055	0.70	0.08	0.91
Gain to feed, kg/kg								
Days 0 to 14	0.300	0.271	0.330	0.286	0.026	<0.01	0.05	0.53
Days 0 to 90	0.127^z^	0.163^y^	0.131^z^	0.175^x^	0.004	<0.01	<0.01	0.07
Days 0 to 97	0.118	0.167	0.121	0.173	0.004	<0.01	0.02	0.46
NEm^3^, Mcal/kg DM	1.28^c^	1.66^b^	1.28^c^	1.70a	0.018	<0.01	0.08	0.05
NEg^3^, Mcal/kg DM	0.72^c^	1.04^b^	0.71^c^	1.08a	0.016	<0.01	0.08	0.05
Water usage^4^, L/hd/d	44.9	39.8	40.0	34.7	1.02	<0.01	<0.01	0.92

^1^ADLIB = formulated to provide 0.99 Mcal NEg/kg DM and fed for ad libitum intake; LIM = formulated to provide 1.32 Mcal NEg/kg of DM and fed at 2.2% of BW (DM basis) daily.

^2^Largest SEM reported.

^3^Net energy for maintenance (NEm) and net energy for gain (NEg), calculated as described by Galyean (2019) based on NRC (1996) requirements.

^4^Total pen water usage ÷ pen head count.

^a,b,c^Within row, means with unlike superscripts differ (*P* ≤ 0.05).

^x,y,z^Within row, means with unlike superscripts tend to differ (*P* ≤ 0.10).

## Effect of Diet

BWs at the completion of the 90-d feeding period were greater (*P *< 0.01; Table 5) for ADLIB compared with LIM; however, following the gut-fill equilibration period, BW was lesser (*P* < 0.01) for ADLIB heifers compared with heifers previously fed LIM. Different responses between ADLIB and LIM following the gut-fill equilibration period demonstrate how dietary roughage concentrations and feeding regimen affect gut-fill and subsequent BW comparisons between treatments. Because heifers in our experiment were limit-fed a common diet at 2.5% of BW (DM basis) for 7 d prior to final BW measurements, BW differences on day 97 likely reflect true treatment differences independent of changes in gut-fill.

Average daily gain from days 0 to 90 and days 0 to 97 followed a similar pattern to BW. Average daily gain from days 0 to 97 was lesser (*P *< 0.01; Table 5) for ADLIB compared with LIM. These data are in contrast with those reported by Felix et al. (2011) who indicated ADG decreased when feed intake was restricted. In that experiment, dietary energy concentration were not increased when DMI was restricted; thus, the reduction in ADG was a result of reduced energy intake. In limit-fed diets, dietary energy concentrations must be increased to achieve similar or greater weight gains. Spore et al. (2019) observed no difference in final BW or ADG between growing heifers fed a diet formulated to contain 0.99 Mcal of NEg/kg for ad libitum intake or limit-fed a diet formulated to contain 1.32 Mcal of NEg/kg; however, in that experiment, limit-fed heifers were fed at 85% of ad libitum intake compared with 2.2% of BW (DM basis) in our study. Regardless, these data suggest that limit-feeding a more energetically dense diet does not negatively influence weight gains during the growing period compared with feeding a high-roughage diet for ad libitum intake.

Gain-to-feed from days 0 to 97 was greater (*P* < 0.01; Table 5) for LIM compared with ADLIB. Improved G:F in LIM was associated with less DMI (*P* < 0.01) and greater (*P* < 0.01) ADG for LIM compared with ADLIB. Similar to our experiment, Scilacci et al. (2024) reported that G:F was 35% greater for heifers limit-fed a high-energy diet at 2.2% of BW (DM basis) compared with heifers fed a high-roughage diet fed for ad libitum intake. Wagner et al. (1990) and Spore et al. (2019) also reported improvement in feed efficiency when growing beef cattle were limit-fed a high-energy diet compared with a high-roughage diet fed for ad libitum intake.

### Activity and Rumination

**Figure 1.**
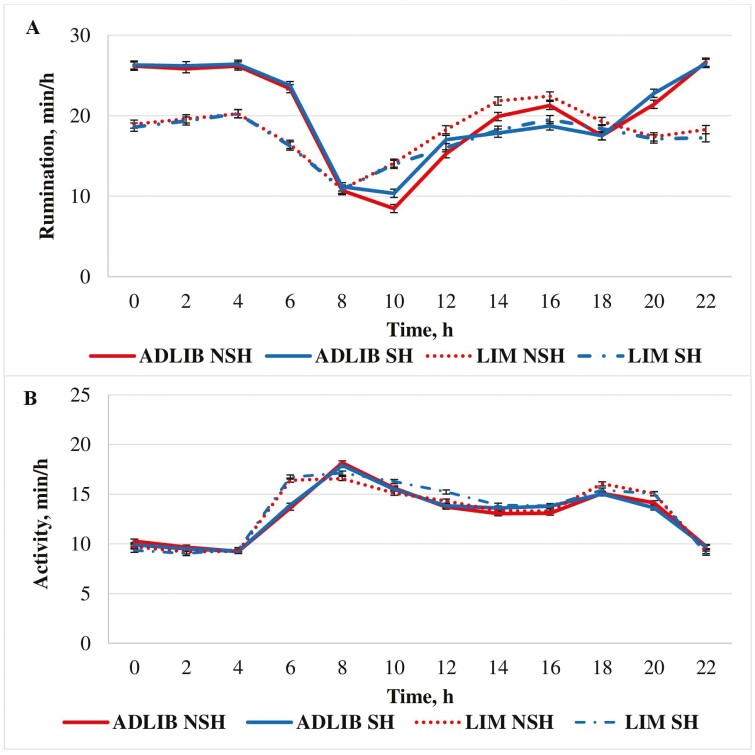
Effect of feeding strategy and shade on average heifer activity and rumination during a 24-h period. ADLIB = formulated to provide 0.99 Mcal NEg/kg DM and fed for ad libitum intake; LIM = formulated to provide 1.32 Mcal NEg/kg of DM and fed at 2.2% of BW (DM basis) daily; SH = provided shade; NSH = not provided shade. Rumination (A): Diet (*P *<* *0.01), Shade (*P *=* *0.31), diet × shade (*P *= 0.17), hour (*P *< 0.01), hour × shade (*P *< 0.01), hour × diet (*P *<* *0.01) hour × diet × shade (*P *= 0.55); SEM = 0.51. Activity (B): diet (*P *= 0.04), shade (*P *=* *0.39), diet × shade (*P *= 0.43), hour (*P *< 0.01), hour × shade (*P *= 0.18), hour × diet (*P *<* *0.01), hour × diet × shade (*P *=* *0.65); SEM = 0.21.

Rumination was greater (*P *< 0.01; Fig. 1) for ADLIB compared with LIM heifers. A diet × hour interaction was observed where ADLIB ruminated more (*P *< 0.01; Fig. 1) compared with LIM from 2000 to 0600 hours. ADLIB ruminated less (*P *< 0.01) compared with LIM at 1000 hours. Differences in time spent ruminating between ADLIB and LIM were likely associated with feed intake and roughage inclusion of each diet. Welch and Smith (1969) reported that rumination time increased as hay intake increased. Similarly, Jennings et al. (2020) observed an increase in rumination time as cornstalk inclusion in the diet increased while Carvalho et al. (2020) determined that rumination time was greater in roughage-based diets compared with grain-based diets. Feeding larger quantities of a diet that contained more roughage likely contributed to the increase in rumination time for ADLIB heifers compared with LIM heifers.

Overall, activity was lesser (*P *= 0.04; Fig. 1) for ADLIB compared with LIM. In addition, LIM heifers were notably more active than ADLIB at 0600 hours (*P *< 0.01; Fig. 1). These data are in agreement with Scilacci et al. (2024) who reported that heifers fed a high-roughage diet for ad libitum intake were less active than heifers limit-fed a high-energy diet at 2.2% of BW (DM basis). In populations of limit-fed animals with greater activity prior to feeding, it may be easier to detect less active or lethargic (i.e., potentially sick) animals. The use of limit-feeding to improve disease detection and management has previously been documented (Galyean et al., 1999). Because limit-fed heifers in our experiment were more active than heifers fed for ad libitum intake, our data suggest that sick or lethargic cattle in the pen may be easier to identify.

### Water Usage

Water usage was greater (*P *< 0.01) for ADLIB compared with LIM and was likely associated with DMI. Winchester and Morris (1956), Mader and Davis (2004), and Ahlberg et al. (2018) reported that water intake increased as DMI increased. In our experiment, heifers fed for ad libitum intake consumed approximately 2.5 kg of DM/d more (*P* < 0.01) than limit-fed heifers. Greater DMI by ADLIB likely led to greater water usage compared LIM. On average, ADLIB consumed approximately 5 L water/d more than LIM.

### Animal Comfort

Panting scores (Table 6) measured at 0930, 1330, 1730 hours, and overall were less (*P* ≤ 0.05) for LIM compared with ADLIB. Lesser panting scores for LIM compared with ADLIB were likely associated with the roughage concentration of each diet and DMI. Achmadi et al. (1994) measured heat production in sheep-fed diets containing differing roughage concentrations and reported that heat production increased as dietary roughage concentrations increased, especially during periods of high environmental heat load. Holt et al. (2004) also evaluated the effects of DMI during periods of heat stress and the subsequent impact on respiration rates and rectal temperatures. In that experiment, steers fed for ad libitum intake had greater respiration rates and rectal temperatures compared with steers fed at 85% of ad libitum intake. Lesser dietary roughage in conjunction with reduced DMI in LIM likely resulted in less heat production from ruminal fermentation compared with ADLIB; therefore, limit-feeding may be a strategy to reduce heat-stress and improve animal comfort during periods of elevated THI.

**Table 6.** Effect of feeding strategy and shade on mean panting scores during periods of heat stress^1^

	Treatment^2^							
	No shade		Shade			*P*-value		
Observation time, hours	ADLIB	LIM	ADLIB	LIM	SEM^3^	Diet	Shade	D × S
0930	0.47	0.41	0.24	0.18	0.037	<0.01	<0.01	0.70
1330	2.15	2.13	1.20	1.12	0.069	0.05	<0.01	0.31
1730	2.13	2.08	1.20	1.13	0.064	<0.01	<0.01	0.65
Overall	1.59	1.54	0.87	0.81	0.051	<0.01	<0.01	0.57

^1^Panting score evaluation adapted from Gaughan et al. (2008).

^2^ADLIB = formulated to provide 0.99 Mcal NEg/kg DM and fed for ad libitum intake; LIM = formulated to provide 1.32 Mcal NEg/kg of DM and fed at 2.2% of BW (DM basis) daily.

^3^Largest SEM reported.

## Effect of Shade

Body weights on days 90 and 97 were greater (*P *< 0.01; Table 5) for SH compared with NSH. Similarly, ADG from days 0 to 97 was greater (*P *< 0.01) for SH compared with NSH. Additionally, G:F from days 0 to 97 was greater (*P *= 0.02) for SH compared with NSH. Because shaded heifers had greater BW on day 90, DMI during the gut-fill equilibration period tended to be greater (*P* = 0.08) for SH compared with NSH; however, the difference was small (0.10 kg of DM/d) and likely did not influence BW on day 97.

Similar to our experiment, Sullivan et al. (2011) reported improvements in final BW, ADG, and G:F in heifers that provided shade compared with heifers not provided shade. Mader et al. (1999), however, indicated growth performance was similar between shaded and nonshaded steers over a 3-yr experiment. Meanwhile, Hagenmaier et al. (2016) observed no differences in growth performance between shaded and nonshaded steers and heifers; however, DMI was greater for shaded heifers compared with nonshaded heifers. Differences in the response to providing shade on growth performance in these experiments may be confounded with environmental factors such as THI and days above a THI of 74. In our experiment, THI averaged 76 in both years which was greater than that reported by Mader et al. (1999) and Hagenmaier et al. (2016). Because heat-stress conditions were present in our experiment, shaded heifers may have experienced a lower heat load and spent less energy dissipating excess heat. Both factors likely contributed to the improvements in growth performance and feed efficiency observed in shaded heifers compared with nonshaded heifers.

### Activity and Rumination

Overall time spent ruminating did not differ (*P* = 0.31; Fig. 1) between shaded and nonshaded heifers. A shade × hour interaction (*P *< 0.01; Fig. 1) was observed where SH ruminated less than NSH from 1400 to 1600 hours; however, time spent ruminating did not differ between SH and NSH heifers at any other time point. Brscic et al. (2007) found cattle exposed to THI above 78 had greater rumination times compared with cattle exposed to THI below 72. In that experiment, authors suggested that cattle ruminated in the standing position during periods of heat stress to help dissipate heat. In our experiment, nonshaded heifers may have experienced temporal patterns of heat stress that contributed to the greater rumination from 1400 to 1600 hours. Despite small differences in time spent ruminating, heifer activity did not differ (*P* = 0.39; Fig. 1) between shaded and nonshaded heifers.

### Water Usage

Water usage was less (*P *< 0.01; Table 5) for SH compared with NSH. Arias and Mader (2011) reported a reduction in water intake for cattle provided shade compared with cattle not provided shade. In our experiment, providing shade likely reduced the heat load of cattle during periods of heat stress. As a result, water loss from increased panting and respiration during periods of heat stress may have been less for SH heifers compared with NSH heifers. On average, SH heifers consumed approximately 5 L water/d less than NSH heifers.

### Animal Comfort

Panting scores measured at 0930, 1330, 1730 hours and overall were less (*P* < 0.01) for SH compared with NSH. Sullivan et al. (2011) reported that panting scores during periods of heat stress were less for shaded heifers compared with nonshaded heifers. Similarly, Mitlöhner et al. (2001) indicated that respiration rates were less for shaded heifers compared with nonshaded heifers. Providing shade during periods of heat stress reduces the animals’ exposure to sunlight. As a result, less heat is accumulated by the animal which decreases the need for panting to expel excess heat.

### Dietary Net Energy Concentrations

A diet × shade interaction (*P* = 0.05) was observed for performance-based NEm and NEg calculations where dietary NEm and NEg were greatest for SH LIM, intermediate for NSH LIM, and least for SH ADLIB and NSH ADLIB. Differences between ADLIB and LIM were expected based on the composition of each diet; however, providing shade to limit-fed heifers led to more efficient utilization of dietary net energy. Providing shade to limit-fed heifers reduced respiration rates and heat stress which likely contributed to improvements in dietary net energy utilization. Overall, dietary NEm and NEg concentrations calculated from performance were less than those calculated using tabular values (NASEM, 2016). Heat stress conditions were present throughout the duration of the study which may have contributed to performance-based dietary net energy concentrations being less than tabular values.

## Experiment 2—Feed Intake, Apparent Diet Digestibility, and Ruminal Fermentation Characteristics

Few diet × shade interactions were observed in Exp. 2. Therefore, the main effects of diet and shade are discussed with those few diet × shade interactions included with discussion of the main effects of diet.

## Effect of Diet

**Figure 2.**
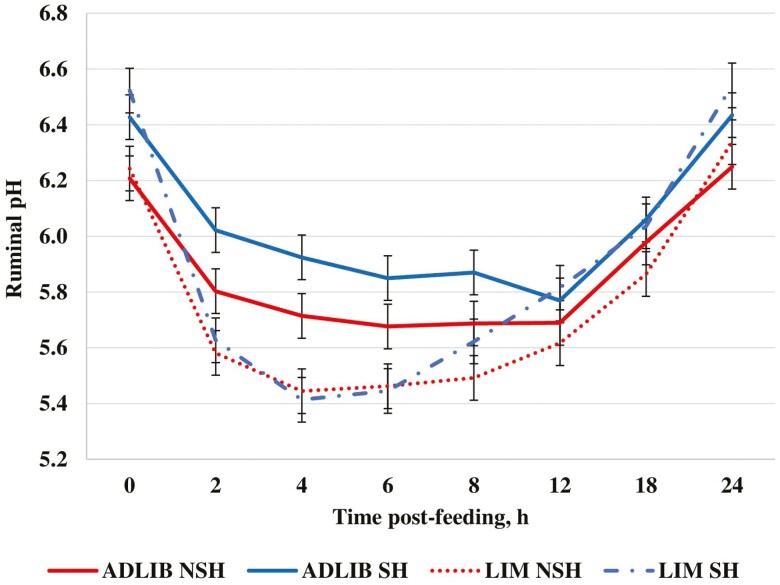
Effect of feeding strategy and shade on ruminal pH of growing heifers during periods of heat stress. ADLIB = formulated to provide 0.99 Mcal NEg/kg DM and fed for ad libitum intake; LIM = formulated to provide 1.32 Mcal NEg/kg of DM and fed at 2.2% of BW (DM basis) daily; SH = provided shade; NSH = not provided shade. Diet (*P *= 0.02), shade (*P *<* *0.01), shade × diet (*P *= 0.71), hour (*P *< 0.01), diet × hour (*P *< 0.01), shade × hour (*P *= 0.46) diet × shade × hour (*P *= 0.44). SEM = 0.08.

By design DM and OM intake (Table 7) were greater (*P* < 0.01) for ADLIB compared with LIM. In addition, NDF and ADF intake were greater (*P *< 0.01) for ADLIB compared with LIM. Greater intakes of NDF and ADF in ADLIB were a result of greater roughage inclusion in ADLIB compared with LIM. Apparent DM and OM digestibilities (Table 7) were greater (*P *< 0.01) for LIM compared with ADLIB, whereas apparent NDF and ADF digestibilities were greater (*P* ≤ 0.04) for ADLIB compared with LIM. Differences in apparent NDF and ADF digestibilities between diets may be associated with differences in dietary ingredients as well as ruminal pH. Ruminal pH was greater (*P *= 0.02; Table 8) for ADLIB compared with LIM. In addition, a diet × hour interaction (*P* < 0.01; Fig. 2) was observed where ruminal pH was greater for ADLIB compared with LIM at 2 to 8 h after feeding. At higher pH fibrolytic bacteria are more active and prevalent; therefore, the ADLIB diet, which had greater ruminal pH, may have increased activity of fiber-digesting bacteria which may have led to improved fiber digestibility. Lana et al. (1998) reported that ruminal pH was more acidic when a 90% concentrate diet was fed compared with 55% or 100% forage-based diets. These data are also in agreement with Spore et al. (2019) and Scilacci et al. (2024) who indicated ruminal pH declined as the concentration of dry-rolled corn in the diet increased.

**Table 7.** Effect of feeding strategy and shade on intake and apparent diet digestibility rate during periods of heat stress

	Treatment^1^							
	No shade		Shade			*P*-value		
Item	ADLIB	LIM	ADLIB	LIM	SEM^2^	Diet	Shade	D × S
Number of observations	15	15	16	16				
Intake, kg/d								
Dry matter	7.64	6.40	7.63	6.35	0.254	<0.01	0.81	0.87
Organic matter	7.08	6.03	7.07	5.98	0.237	<0.01	0.82	0.87
Neutral detergent fiber	3.11	1.67	3.10	1.66	0.091	<0.01	0.84	0.99
Acid detergent fiber	1.55	0.62	1.55	0.61	0.043	<0.01	0.86	0.96
Apparent total tract digestibility, %								
Dry matter	74.10	76.77	72.93	77.36	0.929	<0.01	0.68	0.22
Organic matter	76.70	79.41	75.76	80.10	0.890	<0.01	0.86	0.24
Neutral detergent fiber	71.22	67.11	69.46	68.32	1.414	0.04	0.83	0.23
Acid detergent fiber	68.86	60.87	67.36	63.00	1.666	<0.01	0.95	0.31

^1^ADLIB = formulated to provide 0.99 Mcal NEg/kg DM and fed for ad libitum intake; LIM = formulated to provide 1.32 Mcal NEg/kg of DM and fed at 2.2% of BW (DM basis) daily.

^2^Largest SEM reported.

**Table 8.** Effect of feeding strategy and shade on ruminal fermentation characteristics during periods of heat stress

	Treatment^1^							
	No Shade		Shade			*P*-value		
Item,	ADLIB	LIM	ADLIB	LIM	SEM^2^	Diet	Shade	D × S
Number of observations	15	15	16	16				
Ruminal pH^3^	5.86	5.71	5.99	5.89	0.08	0.02	<0.01	0.71
Ruminal ammonia^3^, m*M*	5.57	4.73	5.01	4.52	0.34	<0.01	0.07	0.40
Liquid passage rate^4^, %/h	8.93	5.15	9.65	5.56	0.463	<0.01	0.19	0.71
Ruminal liquid volume, L	46.95	40.86	42.86	41.41	3.767	0.25	0.62	0.49
Ruminal VFA^3^, m*M*								
Acetate	56.6	49.7	56.7	48.3	1.48	<0.01	0.54	0.44
Propionate	24.5^c^	31.0^a^	23.9^c^	26.8^b^	1.19	<0.01	<0.01	0.02
Butyrate	12.0	11.0	11.9	10.5	0.51	<0.01	0.43	0.60
Valerate	2.16	2.53	2.00	2.15	0.14	<0.01	0.01	0.27
Isobutyrate	0.78	0.80	0.77	0.81	0.03	0.17	0.86	0.88
Isovalerate	1.40	1.64	1.43	1.69	0.12	<0.01	0.60	0.94
Acetate:propionate, mol/mol	2.41^z^	1.74^x^	2.46^z^	1.97^y^	0.07	<0.01	<0.01	0.08
Total VFA	97.1	96.7	96.5	90.5	2.77	0.09	0.08	0.15
Ruminal VFA^3^, molar % of total								
Acetate	58.2^x^	52.1^z^	58.8^x^	54.2^y^	0.62	<0.01	<0.01	0.07
Propionate	25.2^c^	31.5^a^	24.8^c^	29.8^b^	0.75	<0.01	<0.01	0.03
Butyrate	12.1	11.4	12.0	11.8	0.46	0.11	0.60	0.42
Valerate	2.20	2.49	2.04	2.27	0.11	<0.01	0.01	0.62
Isobutyrate	0.82^z^	0.84^z^	0.81^z^	0.93^y^	0.04	<0.01	0.18	0.07
Isovalerate	1.49	1.68	1.58	1.89	0.11	<0.01	0.15	0.52

^1^ADLIB = formulated to provide 0.99 Mcal NEg/kg DM and fed for ad libitum intake; LIM = formulated to provide 1.32 Mcal NEg/kg of DM and fed at 2.2% of BW (DM basis) daily.

^2^Largest SEM reported.

^3^Average values collected at 0, 2, 4, 6, 8, 12, 18, 24 h post-feeding.

^4^Calculated from samples collected a 2, 4, 6, 8, 12, 18 h post-feeding.

^a,b,c^Within row, means with unlike superscripts differ (*P* ≤ 0.05).

^x,y,z^Within row, means with unlike superscripts tend to differ (*P* ≤ 0.10).

**Figure 3.**
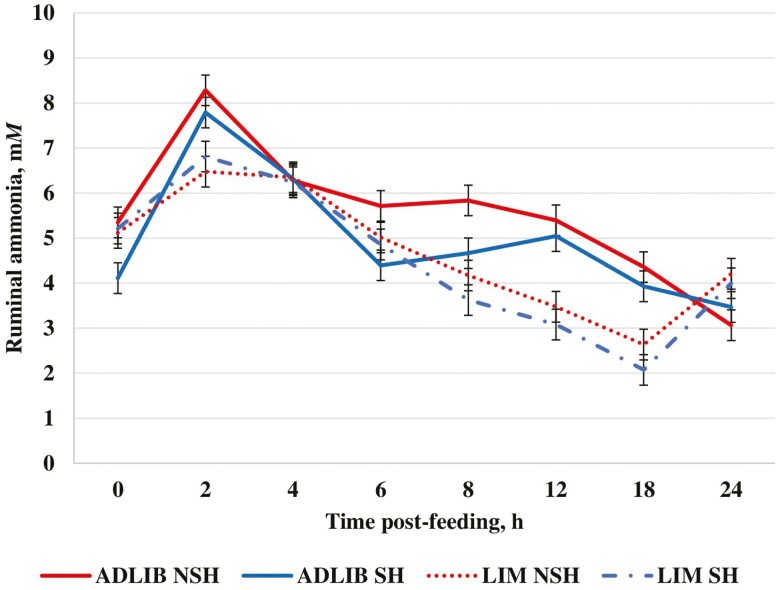
Effect of feeding strategy and shade on ruminal ammonia concentrations of growing heifers during periods of heat stress. ADLIB = formulated to provide 0.99 Mcal NEg/kg DM and fed for ad libitum intake; LIM = formulated to provide 1.32 Mcal NEg/kg of DM and fed at 2.2% of BW (DM basis) daily; SH = provided shade; NSH = not provided shade. Diet (*P *<* *0.01), shade (*P *= 0.07), shade × diet (*P *= 0.40), hour (*P *< 0.01), diet × hour (*P *< 0.01), shade × hour (*P *= 0.84), diet × shade × hour (*P *= 0.77). SEM = 0.34.

Ruminal ammonia concentrations were greater (*P *< 0.01; Table 8) for ADLIB compared with LIM. In addition, a diet × hour interaction (*P *≤* *0.02; Fig. 3) was observed where ADLIB had greater ruminal ammonia concentrations compared with LIM at hours 2, 8, 12, and 18 post-feeding. Differences in ruminal ammonia concentrations were likely associated with dietary crude protein concentrations, ruminal pH, or dietary concentrate level. Dietary crude protein concentrations were 16.3% and 15.4% of diet DM (Table 1) for ADLIB and LIM, respectively. Lana et al. (1998) reported a positive relationship between ruminal pH and ruminal ammonia concentrations. As ruminal pH increases, deamination of amino acids increases. In our experiment, ruminal pH was greater for ADLIB compared with LIM which may have increased amino acid deamination, thus increasing ruminal ammonia concentrations. In addition, LIM contained more fermentable carbohydrates than ADLIB which may have increased ruminal microbial growth and led to greater uptake of ammonia by ruminal microbes.

Total ruminal VFA concentrations (Table 8) tended to be greater (*P* = 0.09) for ADLIB compared with LIM. Spore et al. (2019) and Clark et al. (2007) also reported a reduction in total VFA concentrations when calves were limit-fed compared with calves fed for ad libitum intake. Ruminal acetate concentrations were greater (*P* < 0.01) for ADLIB compared with LIM. In addition, a tendency for a diet × shade interaction (*P *≤* *0.07) was observed for molar proportions of ruminal acetate where they tended to be greatest for SH ADLIB and NSH ADLIB, intermediate for SH LIM and least for NSH LIM. Similarly, diet × shade interactions (*P *≤* *0.03) for ruminal concentrations and molar proportions of ruminal propionate were also observed with responses being inverse of those observed for acetate proportions. Subsequently, a tendency for a diet × shade interaction (*P *= 0.07) was observed for acetate:propionate, which followed the pattern for acetate proportions. Differences in concentrations and proportions of ruminal acetate and propionate for LIM compared with ADLIB were likely associated with diet composition. Bauman et al. (1971) reported that ruminal concentrations of acetate decreased whereas ruminal concentrations of propionate increased as concentrates replaced roughages in diets fed to lactating Holstein cows; therefore, more concentrate and less roughage inclusion in LIM compared with ADLIB likely shifted VFA production from acetate to propionate.

Ruminal butyrate concentrations were greater (*P *< 0.01; Table 8) for ADLIB compared with LIM. Although proportions of ruminal butyrate did not differ (*P* = 0.11) between diets, the proportion of ruminal butyrate followed a trend similar to ruminal butyrate concentrations. Greater roughage inclusion and increased ruminal pH of ADLIB may have increased butyrate-producing bacteria and ultimately increased ruminal butyrate concentrations compared with LIM. Ruminal valerate concentrations and proportions of valerate were lesser (*P *< 0.01) for ADLIB compared with LIM. These data agree with Coe et al. (1999) who reported the proportion of ruminal valerate increased as roughage inclusion in the diet decreased. Isobutyrate concentration did not differ between diets (*P* = 0.17), but proportion of isobutyrate as well as isovalerate concentration and proportion were lesser (*P* < 0.01) for ADLIB compared with LIM.

Liquid passage rate was greater (*P *< 0.01; Table 8) for ADLIB compared with LIM. These results agree with Spore et al. (2019) who observed a linear reduction in ruminal liquid passage rate as feed intake was reduced and dietary energy concentrations were increased. Ruminal liquid volume did not differ (*P *= 0.25) between ADLIB and LIM.

## Effect of Shade

No effect of shade was observed for DM, OM, NDF, or ADF intake or apparent diet digestibility (Table 7; *P *≥ 0.68). Bernabucci et al. (1999) compared diet digestibilities of heifers housed in thermoneutral conditions with those housed in a hot environment (THI = 84) for 40 d. Heifers housed in thermal neutral conditions were transitioned for 3 d into the hot environment where cattle spent 13 d; then they were maintained in the hot environment for an additional 24-d trial. Dry matter intake was less for heifers housed in a hot environment compared with those in thermoneutral conditions. The authors also observed an increase in diet digestibility when cattle were placed in the hot environment for 13 d, then during the final 4-d trial digestibility returned to the initial level observed when cattle were housed in thermoneutral conditions. These data suggest cattle can adapt to some extent during hot conditions which could possibly explain our observations in diet digestibility. Dry matter intake was not affected by shade in this experiment, and this may have limited our ability to observe differences in diet digestibility.

Ruminal pH was greater (*P *< 0.01; Table 8; Fig. 2) for SH compared with NSH. During periods of heat stress, blood flow is shifted from the gastrointestinal tract to the extremities to aid in heat loss (Lees et al., 2019). The shift in blood flow that occurs during periods of heat stress could potentially decrease absorption of VFA causing an observed reduction in ruminal pH in NSH heifers compared with SH heifers. Ruminal ammonia concentration tended to be lesser (*P *= 0.07; Table 8; Fig. 3) for SH compared with NSH. At lower ruminal pH, ammonia absorption rate would decrease due to increased protonation of ruminal ammonia (Lana et al., 1998). This could lead to a higher concentration of ruminal ammonia in NSH heifers compared with SH heifers.

**Figure 4.**
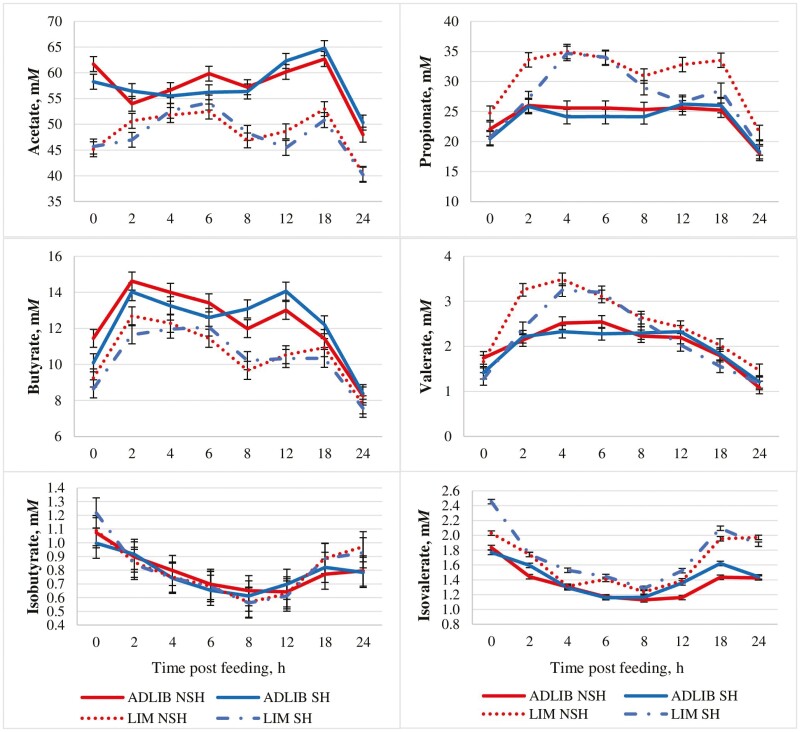
Effect of feeding strategy and shade on ruminal volatile fatty acid concentrations of growing heifers during periods of heat stress. ADLIB = formulated to provide 0.99 Mcal NEg/kg DM and fed for ad libitum intake; LIM = formulated to provide 1.32 Mcal NEg/kg of DM and fed at 2.2% of BW (DM basis); SH = provided shade; NSH = not provided shade. Acetate: diet (*P *< 0.01), shade (*P *= 0.54), diet × shade (*P *= 0.44), hour (*P *< 0.01), diet × hour (*P *< 0.01), shade × hour (*P *= 0.94), diet × shade × hour (*P *= 0.41); SEM = 1.45. Propionate: diet (*P *< 0.01), shade (*P *< 0.01), diet × shade (*P *= 0.02), hour (*P *< 0.01), diet × hour (*P *< 0.01), shade × hour (*P *= 0.94), diet × shade × hour (*P *= 0.30); SEM = 1.20. Butyrate: diet (*P *< 0.01), shade (*P *= 0.43), diet × shade (*P *= 0.60), hour (*P *< 0.01), diet × hour (*P *= 0.20), shade × hour (*P *= 0.64), diet × shade × hour (*P *= 0.76); SEM = 0.50. Valerate: diet (*P *<* *0.01), shade (*P *= 0.01), diet × shade (*P *= 0.27), hour (*P *< 0.01), diet × hour (*P *< 0.01), shade × hour (*P *= 0.88), diet × shade × hour (*P *= 0.02); SEM = 0.14. Isobutyrate: diet (*P *<* *0.01), shade (*P *= 0.86), diet × shade (*P *= 0.88), hour (*P *< 0.01), diet × hour (*P *< 0.01), shade × hour (*P *= 0.89), diet × shade × hour (*P *= 0.41); SEM = 0.03. Isovalerate: diet (*P *<* *0.01), shade (*P *= 0.60), diet × shade (*P *= 0.94), hour (*P *< 0.01), diet × hour (*P *= 0.01), shade × hour (*P *= 0.89), diet × shade × hour (*P *= 0.31); SEM = 0.11.

Total VFA concentrations tended to be lesser (*P* = 0.08; Table 8) for SH compared with NSH. Ruminal concentrations and proportions of butyrate, isobutyrate, and isovalerate did not differ (*P *≥ 0.15) between SH and NSH; however, ruminal concentrations and proportions of valerate were greater (*P* = 0.01) for NSH compared with SH. There were no shade × hour interactions for concentrations of any of the individual VFA (*P *≥ 0.64; Fig. 4).

Liquid passage rate and ruminal liquid volume did not differ (*P* ≥ 0.19) between NSH and SH. Providing shade during periods of heat stress can increase DMI in animals fed for ad libitum intake which could influence liquid passage rate and ruminal liquid volumes (Mitlöhner et al., 2001). There were no differences in intake between shaded treatments which may explain the lack of differences in liquid passage rate and ruminal liquid volume.

## Conclusions

Minimization of heat load experienced by growing calves during summer months is beneficial from both a performance and animal comfort perspective. Implementation of shade improved heifer final BW, ADG, and feed efficiency during a 2-yr summer trial. In addition, water usage and panting scores were reduced when heifers were provided access to shade. Limit feeding a high-energy diet improved feed efficiency, apparent diet digestibility, and animal comfort and subsequently reduced water usage compared with feeding a high-roughage diet for ad libitum intake. When applied together, feed efficiency was 46.6% greater while water usage 22.7% less in limit-fed shaded heifers compared with nonshaded heifers fed for ad libitum intake. Overall, these data suggest that limit feeding and providing shade are heat-stress mitigation strategies that can be used to improve animal performance, water usage, and animal comfort.

## Supplementary Data

Supplementary data are available at *Translational Animal Science* online.

txae161_suppl_Supplementary_Appendix

## Abbreviations:

ADF: acid detergent fiber
ADG: average daily gain
AIC: Akaike information criterion
BW: body weight
CIS: clinical illness score
DM: dry matter
DMI: dry matter intake
G:F: gain:feed
NDF: neutral detergent fiber
NEg: net energy for gain
NEm: net energy for maintenance
OM: organic matter
THI: temperature humidity index
VFA: volatile fatty acid
