# Supplementary material for: Effect of shade and limit feeding in growing beef heifers during periods of heat stress
Source: Transl Anim Sci. 2024 Nov 19;8:txae161. doi: 10.1093/tas/txae161 (PMC11630847; doi:10.1093/tas/txae161)
Supplement: txae161_suppl_Supplementary_Appendix [file txae161_suppl_supplementary_appendix.docx]

| **Appendix 1**. Effect of feeding strategy and shade on heifer performance during periods of heat stress | | | | | | | | | | | | | | | |
| --- | --- | --- | --- | --- | --- | --- | --- | --- | --- | --- | --- | --- | --- | --- | --- |
|  | Treatment^1^ | | | | | | | |  |  |  |  |  |  |  |
|  | Year 1 | | | | |  | Year 2 | | | | |  |  |  |  |
|  | No shade | |  | Shade | |  | No shade | |  | Shade | |  | *P*-value^3^ | | |
| Item, | ADLIB | LIM |  | ADLIB | LIM |  | ADLIB | LIM |  | ADLIB | LIM | SEM^2^ | D × Y | S × Y | D × S× Y |
| Body weight, kg |  |  |  |  |  |  |  |  |  |  |  |  |  |  |  |
| d 0 | 256 | 256 |  | 254 | 255 |  | 247 | 247 |  | 246 | 246 | 5.8 | 0.35 | 0.80 | 0.97 |
| d 14 | 281 | 278 |  | 282 | 277 |  | 274 | 266 |  | 274 | 267 | 6.4 | 0.03 | 0.74 | 0.28 |
| d 90 | 368 | 367 |  | 375 | 370 |  | 345 | 336 |  | 354 | 347 | 8.2 | 0.16 | 0.09 | 0.38 |
| d 97 | 367 | 379 |  | 372 | 380 |  | 348 | 348 |  | 358 | 357 | 7.9 | < 0.01 | 0.03 | 0.73 |
| Average daily gain, kg/d |  |  |  |  |  |  |  |  |  |  |  |  |  |  |  |
| d 0-14 | 1.78 | 1.57 |  | 1.99 | 1.54 |  | 1.86 | 1.34 |  | 2.00 | 1.51 | 0.202 | 0.15 | 0.61 | 0.28 |
| d 0-90 | 1.25 | 1.23 |  | 1.34 | 1.27 |  | 1.08 | 0.99 |  | 1.20 | 1.12 | 0.054 | 0.30 | 0.07 | 0.37 |
| d 0-97 | 1.15 | 1.26 |  | 1.21 | 1.28 |  | 1.04 | 1.05 |  | 1.16 | 1.15 | 0.050 | < 0.01 | 0.02 | 0.71 |
| Dry matter intake, kg/d |  |  |  |  |  |  |  |  |  |  |  |  |  |  |  |
| d 0-14 | 6.16 | 5.53 |  | 6.12 | 5.51 |  | 6.08 | 5.20 |  | 6.17 | 5.20 | 0.154 | < 0.01 | 0.33 | 0.47 |
| d 0-90 | 9.45^ab^ | 7.07^c^ |  | 9.70^a^ | 7.06^c^ |  | 8.91^b^ | 6.48^c^ |  | 9.86^a^ | 6.58^c^ | 0.250 | < 0.01 | < 0.01 | 0.03 |
| d 0-97 | 9.45^ab^ | 7.24^c^ |  | 9.69^a^ | 7.23^c^ |  | 9.69^b^ | 6.62^c^ |  | 9.78^a^ | 6.73^c^ | 0.241 | < 0.01 | < 0.01 | 0.03 |
| d 90-97 | 9.45 | 9.40 |  | 9.53 | 9.52 |  | 9.42 | 9.43 |  | 9.54 | 9.50 | 0.077 | 0.91 | 0.99 | 0.67 |
| Gain to feed, kg/kg |  |  |  |  |  |  |  |  |  |  |  |  |  |  |  |
| d 0-14 | 0.293 | 0.285 |  | 0.330 | 0.284 |  | 0.306 | 0.257 |  | 0.327 | 0.288 | 0.0368 | 0.44 | 0.72 | 0.28 |
| d 0-90 | 0.132^c^ | 0.174^a^ |  | 0.138^c^ | 0.179^a^ |  | 0.122^c^ | 0.152^b^ |  | 0.123^c^ | 0.170^a^ | 0.0062 | < 0.01 | 0.07 | 0.05 |
| d 0-97 | 0.121 | 0.174 |  | 0.124 | 0.177 |  | 0.114 | 0.159 |  | 0.118 | 0.169 | 0.0054 | 0.22 | 0.33 | 0.46 |
| NEm^3^, Mcal/kg DM | 1.30 | 1.70 |  | 1.31 | 1.71 |  | 1.27 | 1.62 |  | 1.26 | 1.68 | 0.026 | 0.34 | 0.62 | 0.07 |
| NEg^3^, Mcal/kg DM | 0.73 | 1.08 |  | 0.74 | 1.09 |  | 0.71 | 1.01 |  | 0.70 | 1.06 | 0.022 | 0.34 | 0.62 | 0.07 |
| Water usage^4^, L/hd/d | 44.7 | 40.4 |  | 39.7 | 35.6 |  | 45.2 | 39.2 |  | 40.3 | 33.7 | 1.48 | 0.23 | 0.84 | 0.80 |
| ^1^ ADLIB = formulated to provide 0.99 Mcal NEg/kg DM and fed for ad libitum intake; LIM = formulated to provide 1.32 Mcal NEg/kg of DM and fed at 2.2% of BW (DM basis) daily.  ^2^ Largest SEM reported.  ^3^ D = Diet; S = Shade; Y = Year  ^a,b,c^ Within row, means with unlike superscripts differ (*P* ≤ 0.05). | | | | | | | | | | | | | | | |
